# From Awareness to Practice: How Parents Seek Support for Children’s Sexuality Education

**DOI:** 10.3390/children13101378

**Published:** 2026-10-11

**Authors:** Kyungin Cheon, Sujin Lee

**Affiliations:** 1 Jeju Institute of Public Health & Health Policy, Jeju 63241, Republic of Korea; kicheon0804@gmail.com; 2 College of Nursing, Dongguk University-WISE, Gyeongju 21936, Republic of Korea

**Keywords:** sexuality education, parents, child, preschool, information seeking behavior, mixed methods research

## Abstract

**Highlights:**

**What are the main findings?**

- Parents’ sexuality education developed through four interconnected domains, from recognizing sexuality in everyday situations to seeking structured and collaborative support.
- Parents relied heavily on storybooks and online information to manage immediate concerns, revealing a persistent gap between recognizing their educational responsibility and being able to implement sexuality education.

**What are the implications of the main findings?**

- Parents need developmentally appropriate, practical guidance that supports them in responding to children’s sexuality-related questions and everyday teachable moments.
- Pediatric and community health systems should provide structured, family-centered resources that strengthen parents’ capacity rather than treating sexuality education as an individual parental responsibility.

**Abstract:**

**Background/Objectives**: This study aimed to explore parents’ responses and information-seeking patterns regarding early childhood sexuality education by integrating semantic network analysis of digital discourse with in-depth interviews. **Methods**: A mixed-methods design was employed. Semantic network analysis was conducted on 124 online posts from Korean parenting communities and Q&A platforms, while in-depth interviews were conducted with 18 parents of young children. Online data were analyzed using semantic network analysis, and interview data were analyzed using inductive thematic analysis. **Results**: Four interconnected domains of the parental response were identified: (1) recognizing sexuality in everyday contexts, (2) adopting initial coping strategies, (3) struggling with the role–action gap, and (4) seeking structured and collaborative support. Although parents recognized their role as primary sexuality educators, they experienced substantial uncertainty about how to respond and relied heavily on mediated resources, particularly sexuality education books and online information. **Conclusions**: Parents need practical, developmentally appropriate guidance to respond confidently to children’s sexuality-related questions and everyday situations. Family-centered support systems and accessible, evidence-based resources should be developed to strengthen parents’ capacity and bridge the gap between recognizing their educational responsibility and implementing sexuality education in daily life.

## 1. Introduction

Early childhood is a period of rapid cognitive development and increasing curiosity [1]. In particular, children aged 4–6 years show curiosity about their own and others’ bodies, recognize physical differences, and gradually develop an awareness of privacy [2,3]. Such interest in and exploration of the body are part of normative development in early childhood; however, children’s understanding of appropriate physical boundaries remains limited at this age [2]. Therefore, early childhood represents an important developmental period for sexuality education, during which developmentally appropriate guidance can help children understand their own bodies, develop a sense of body ownership, respect their own and others’ bodies, and develop an understanding of appropriate physical boundaries and body safety [2].

Children’s understanding of sexuality is shaped not only through systematic education provided in kindergartens or childcare centers but also through interactions with people around them in everyday life [2,4]. In particular, children may construct their own knowledge about sexuality based on fragmented information they encounter in their surroundings, and in this process, they may acquire misinformation or stereotypes [4]. Therefore, early childhood sexuality education should not be limited to one-time instruction at a specific time or place but should occur continuously through various situations that naturally arise in daily life, such as when children show interest in their own or others’ bodies or ask related questions [2,4]. In these situations, communication should be appropriate to the child’s age and developmental level, supporting their understanding of body ownership, physical boundaries, and body safety [2].

As primary caregivers, parents are key agents of sexuality education within the family because they directly respond to children’s sexuality-related questions or behaviors in everyday life [5]. However, many parents lack the knowledge and preparation needed to provide sexuality education appropriate to their child’s age and developmental level [5,6] and experience uncertainty and difficulty in explaining and communicating sexuality-related information to their children [5,7].

Previous research on early childhood sexuality education has documented these challenges by examining parents’ knowledge and perceptions, the extent and difficulties of providing sexuality education at home, and their educational and informational needs [5,6,8,9]. Collectively, these studies indicate that parents need specific and reliable information and guidance to provide age- and situation-appropriate sexuality education [5,8,9]. However, while previous research has contributed to identifying parents’ knowledge, perceptions, difficulties, and information needs, relatively few studies have examined how parents respond to sexuality-related situations that arise in everyday life and how they seek the information they need in the process.

Computational analysis of online discourse has been used to identify patterns and topics in sexuality education discussions [10], and online posts can provide insight into spontaneously expressed questions and information needs [11,12,13]. In-depth interviews can complement these data by providing contextual insight into how parents interpret and respond to sexuality-related situations in their everyday lives [14]. Therefore, this study integrated analysis of Korean online discourse with one-on-one in-depth interviews to explore how parents respond to everyday sexuality-related situations and seek the information they need in the context of early childhood sexuality education.

## 2. Materials and Methods

### 2.1. Design

This study employed a mixed-methods design, in which text network analysis and one-on-one in-depth interviews were conducted in parallel. Online textual data were analyzed to identify patterns of co-occurrence among key terms. Qualitative data from interviews were analyzed using an inductive thematic analysis approach following Braun and Clarke [15] to explore participants’ interpretations and meaning-making processes. The findings from both strands were integrated during the interpretation domain to provide a comprehensive understanding of the phenomenon.

### 2.2. Study Setting and Recruitment

This study utilized a mixed-method approach, combining the analysis of online posts with one-on-one in-depth interviews to capture a comprehensive landscape of parental experiences.

For the digital component, data were collected from major domestic parenting communities and Q&A platforms in South Korea, specifically focusing on Naver’s parenting communities and its Q&A service, which are among the most widely used online platforms in Korea [16]. One specific online community (“cafe”) was purposively selected based on its large user base and high level of active interaction. Data collection targeted posts published between 2020 and February 2026.

For the individual component, one-on-one in-depth interviews were conducted with parents to explore lived experiences in depth. Participants were recruited through purposive sampling to include a broad spectrum of perceptions, ranging from those with little interest or no prior experience in sexuality education to those who were highly engaged and actively teaching their children. This inclusive strategy aimed to capture the wide variety of parental lived experiences through individualized interactions.

### 2.3. Inclusion and/or Exclusion Criteria

The research participants were defined based on their role as primary caregivers and the developmental stage of their children. The inclusion criteria for this study were parents or primary caregivers holding the main responsibility for a child’s daily care and upbringing, caregivers raising children between the ages of 4 and 6, and caregivers capable of communicating fluently in Korean to ensure the depth of qualitative data. The age range of 4 to 6 years was established with reference to the developmental stages defined by the World Health Organization (WHO) Regional Office for Europe and the Federal Centre for Health Education (BZgA) [17], as well as the International Technical Guidance on Sexuality Education led by the United Nations Educational, Scientific and Cultural Organization (UNESCO) [18], representing an interval characterized by an increase in children’s sex-related inquiries where parent-led education is most active. Conversely, the exclusion criteria included parents of children who had already entered formal primary schooling (over age 6), as the research specifically focuses on the paramount role of parents in the home setting prior to institutionalized education, and caregivers who were not the primary person responsible for the child’s daily upbringing, such as occasional sitters or extended family members without primary custody. For a detailed breakdown of the participants’ demographic characteristics and their children’s profiles, please refer to the corresponding table in the Section 3.

### 2.4. Data Collection

Online textual data were collected from parenting communities and question-and-answer platforms hosted on Naver [16]. Using the single Korean search term “성교육” (seonggyoyuk; sexuality education), posts were retrieved through a web crawling program implemented in Python (version 3.12.12). No morphological or lexical variants were used as additional search terms. The single search term was selected to retrieve posts explicitly framed by users as relating to sexuality education. Posts published between 2020 and February 2026 were included, and the online data were crawled and extracted in March 2026. The initial retrieval yielded 318 posts from parenting communities and 1001 posts from question-and-answer platforms.

A two-stage screening process was applied. In the first stage, automated screening was performed using Python (version 3.12.12) based on predefined keyword-based exclusion criteria. Keywords indicative of advertisements or promotional content (e.g., marketing, events, free trials, inquiries, applications, recruitment, links, reservations, announcements, or supporter programs) and those indicating non-preschool populations (e.g., upper elementary, middle school, or high school students and adolescents) were used to identify posts for exclusion. This automated screening resulted in the removal of 751 posts, yielding 568 posts for further screening. In the second stage, manual screening was conducted to ensure content relevance. One researcher reviewed all remaining posts and excluded posts that (1) lacked substantive content related to sexuality education (e.g., general daily narratives, attendance confirmations, photo sharing, or event participation records); (2) consisted only of simple book reviews or purchase confirmations without substantive sexuality education content; or (3) did not target preschool-aged children or had unclear target populations. Cases for which the inclusion or exclusion decision was unclear were discussed with another researcher, and the final decision was reached through consensus. After manual screening, a total of 124 posts (84 from the question-and-answer platform and 40 from the parenting community) were retained for analysis. Only publicly accessible posts were included, and no identifiable personal information was collected (Figure 1).

Participants for interviews were recruited through online communities for parents and were provided with information about the study’s purpose regarding parents’ perceptions, experiences, and educational needs concerning early childhood sexuality education. Informed consent was obtained online prior to participation. Interviews were conducted by the primary author (a female assistant professor in nursing holding a PhD, with extensive training and experience in qualitative research). To maintain objectivity, no prior relationship existed between the researcher and the participants. Participants were fully informed of the researcher’s academic background and the study’s objective to explore parental perspectives on sexuality education. Data were collected through one-on-one semi-structured in-depth interviews (Supplementary file S1). Following consent, interviews were conducted either face-to-face or Via video conferencing (e.g., Zoom), depending on participant preference. Each interview lasted approximately 60–90 min. Field notes were also utilized to record non-verbal expressions, gestures, and contextual observations during the interviews. Each participant completed one individual in-depth interview, and everyone participated in the interview. In addition, a re-interview was not conducted. All participants who were recruited and consented to participate completed the interview, and no participants withdrew from the study. All interviews were audio-recorded with participants’ consent and transcribed verbatim for analysis. Data collection continued until data saturation was reached, defined as the point at which no new themes or insights emerged. A total of 18 participants were included in the final analysis.

### 2.5. Data Analysis

The collected online text data were preprocessed and analyzed using Python (version 3.13.15) and KoNLPy (version 0.6.0), a Python-based open-source library for Korean natural language processing [19]. Before morphological analysis, spacing and orthographic variants of selected compound terms were standardized (e.g., “남자 아이” (namja ai; boy) and “남자아이” (namjaai; boy) to “남아” (nama; boy), and “성 교육” (seong gyoyuk; sexuality education) to “성교육” (seonggyoyuk; sexuality education)). Korean morphological analysis was performed using the Okt analyzer, and only nouns were extracted for subsequent analysis. No separate lemmatization procedure was applied. After noun extraction, selected lexical variants representing the same or closely related concepts were standardized to common analytical terms (e.g., “동화” (donghwa; storybook), “동화책” (donghwachaek; storybook), and “그림책” (geurimchaek; picture book) to “성교육도서” (seonggyoyuk doseo; sexuality education books); and “유튜브” (yutyubeu; YouTube), “영상” (yeongsang; video), and “스마트폰” (seumateupon; smartphone) to “미디어” (midieo; media)). The semantic rationale for aggregating the book-related expressions was established through contextual review of the original posts. In the included corpus, these expressions were used in the context of seeking or discussing book-based resources for young children’s sexuality education; therefore, they were standardized to the common analytical category “sexuality education books.” Single-character nouns and predefined stopwords were removed from the analysis (Supplementary file S3). No additional low-frequency cutoff was applied during term-frequency analysis. Following the preprocessing, term-frequency analysis was conducted to identify frequently occurring terms in the online corpus.

Term frequency–inverse document frequency (TF-IDF) scores were calculated using scikit-learn (version 1.6.1) at the document level, with each of the 124 posts treated as a separate document. The mean TF-IDF score for each term was calculated across all 124 posts, and the 20 terms with the highest mean TF-IDF scores were selected as candidate terms for the subsequent network analysis. To identify the relationships among the selected terms, co-occurrence was defined as the occurrence of two terms within the same sentence, with repeated occurrences of the same term within a sentence counted only once. An undirected weighted co-occurrence network was then constructed using NetworkX (version 3.6.1), in which nodes represented terms and edge weights represented the number of sentences in which each pair of terms co-occurred. Only edges with a sentence-level co-occurrence frequency of two or more were retained in the final network. Co-occurrence networks provide a structural representation of the relationships among concepts within a text [20]. Social Network Analysis (SNA) techniques were applied to the constructed network to perform centrality analysis. Specifically, normalized degree centrality, normalized betweenness centrality, and closeness centrality were calculated to determine the importance of key terms and their structural roles within the network [21]. Degree centrality indicates how many other terms a term is directly connected to, reflecting the extent of its direct connections within the network. Betweenness centrality indicates how often a term serves as a bridge connecting other terms or different parts of the network. Closeness centrality indicates how easily a term can reach all other terms through the network, reflecting how centrally it is positioned within the overall network. Centrality measures were calculated based on the network’s connection structure, without considering the strength of the connections (edge weights). The final network formed a single connected component. Louvain community detection was performed on the weighted, undirected network using edge weights, with a resolution of 1.0. The number of communities was not specified in advance. Weighted modularity (Q) was calculated to quantify the strength of the community structure identified by the Louvain algorithm. The resulting communities were interpreted based on their constituent keywords and co-occurrence relationships (Supplementary file S4).

All text preprocessing and computational analyses were conducted using the original Korean text. Korean terms identified through the analyses were translated into English only after the analyses were completed for presentation in the manuscript, tables, and figures. Thus, translation did not affect tokenization, TF-IDF calculation, co-occurrence relationships, or network structure.

Interview data were analyzed using a coding-reliability approach to thematic analysis following the guidelines of Braun and Clarke [15]. All interviews were transcribed verbatim, and the researchers repeatedly reviewed the transcripts to achieve data familiarization. Data analysis was conducted manually without the use of qualitative data analysis software. Initial codes were generated inductively from the data and subsequently grouped into categories based on conceptual similarities. These categories were then developed into themes through an iterative process of reviewing and refining the data. Two researchers independently conducted coding and theme development, and discrepancies were resolved through discussion until consensus was reached. The analysis involved continuous movement between the data and emerging themes to ensure that the findings accurately reflected participants’ experiences. In addition, the identified themes were organized into interconnected domains to explain participants’ responses to their underlying experiences.

The findings from the two data sources were integrated during the interpretation domain through a side-by-side comparison. The two researchers jointly compared the online network communities with the qualitative domains identified from the interviews and examined areas of convergence, complementarity, and divergence across the findings. Through discussion and consensus, corresponding and complementary patterns across the two data sources were mapped to develop the integrated framework presented in the discussion . Because the online posters and interview participants were independent samples, integration was conducted at the pattern level rather than at the individual level.

### 2.6. Ethical Considerations

The online data collection and qualitative interviews were conducted within the scope of the study approved by the Dongguk University Institutional Review Board (DGU IRB 20250043, approved 27 November 2025).

For the one-on-one in-depth interviews, all participants were provided with verbal and written explanations regarding the study’s purpose, the voluntary nature of participation, and the right to withdraw at any time without penalty. Written informed consent was obtained from all interviewees prior to data collection. To ensure anonymity, all personal identifiers were replaced with pseudonyms (e.g., Participant A).

Regarding the online data, only publicly accessible original posts were collected from parenting communities and Q&A platforms. Comments were not collected or analyzed. Data collection was limited to publicly accessible online content and did not involve access to private or password-protected content. Usernames, user IDs, specific location information, or other identifiable personal information were not collected at any stage; therefore, no subsequent anonymization of identifiers was required. No verbatim excerpts from the online posts are presented in this manuscript, and the online findings are reported only in aggregate form to minimize the risk of re-identification. All digital and interview data were stored on a password-protected drive and will be permanently deleted after the mandatory retention period.

### 2.7. Rigor and Reflexivity

To ensure trustworthiness, the criteria of credibility, transferability, dependability, and confirmability were applied [22]. Credibility and dependability were established Via investigator triangulation; two researchers with expertise in social network analysis and qualitative nursing research independently coded the data. The interviews were conducted in Korean, and the quotations presented in the manuscript were translated from Korean into English for publication. The English translations were reviewed and edited to ensure natural and idiomatic expression while preserving the participants’ original meaning and voice. The researchers independently reviewed the transcripts and the English translations of selected quotations to ensure transcription accuracy and semantic equivalence, and any discrepancies were discussed and resolved by consensus. Data collection continued until data saturation was reached.

Confirmability and reflexivity were maintained using a reflexive journal to bracket researchers’ biases. Regular peer debriefing ensured findings remained grounded in participant narratives and online data. Transferability was addressed through thick descriptions of participant characteristics, digital contexts, and the sociocultural background of the study setting (Supplementary file S2).

## 3. Results

### 3.1. Online Text Analysis Results

#### 3.1.1. The Frequencies of Keywords Related to Sexuality Education

In the analysis of the combined online posts, “sexuality education books” was the most frequently occurring keyword (*n* = 129), followed by “recommendation” (*n* = 79), “boy” (*n* = 46), “curiosity” (*n* = 26), and “kindergarten” (*n* = 26). In the Community posts, the five most frequent keywords were “sexuality education books” (*n* = 26), “recommendation” (*n* = 14), “boy” (*n* = 10), “media” (*n* = 9), and “girl” (*n* = 8). In the Q&A posts, the five most frequent keywords were “sexuality education books” (*n* = 103), “recommendation” (*n* = 65), “boy” (*n* = 36), “kindergarten” (*n* = 26), and “curiosity” (*n* = 21) (Table 1).

#### 3.1.2. Keyword Centrality Analysis

Centrality analysis was conducted for the 18 keywords retained in the final co-occurrence network (Table 2). “Sexuality education books” had the highest degree centrality (Cd = 0.765), followed by “recommendation” (Cd = 0.706), “boy” (Cd = 0.647), and “father” (Cd = 0.588). For betweenness centrality, “boy” had the highest value (Cb = 0.262), followed by “sexuality education books” (Cb = 0.225), “media” and “starting point” (Cb = 0.118 each), and “recommendation” (Cb = 0.108). Closeness centrality was highest for “sexuality education books” (Cc = 0.773), followed by “recommendation” and “boy” (Cc = 0.739 each), and “father” (Cc = 0.680). The centrality values for all 18 keywords are presented in Table 2.

#### 3.1.3. Co-Occurrence Network of Keywords

Of the 20 candidate terms selected based on mean TF-IDF scores, “child” and “method” did not form any edges meeting the sentence-level co-occurrence threshold of two or more and were therefore not retained in the final network. The final co-occurrence network consisted of 18 keywords and 57 edges. Louvain community detection identified three communities within the network, with a weighted modularity of Q = 0.164 (Figure 2). The identified communities were interpreted as exploratory structural groupings based on keyword co-occurrence patterns rather than as sharply separated latent categories.

- The first community included “sexuality education books,” “recommendation,” “curiosity,” “starting point,” “book series,” “amount of text,” “audio pen,” “concern,” and “when.” This grouping was interpreted as reflecting discussions related to sexuality education resources and timing.
- The second community comprised “boy,” “girl,” “young child,” “mother,” “father,” “kindergarten,” and “education.” This grouping was interpreted as reflecting parent–child and educational contexts in which sexuality education was discussed.
- The third community consisted of “media” and “help.” This smaller grouping was interpreted as reflecting discussions involving media and help in relation to sexuality education.

### 3.2. One-on-One In-Depth Interviews

#### 3.2.1. Characteristics of Participants and Their Children

A total of 18 participants were included in the study. Most participants were female (*n* = 16, 88.9%) and in their 30s, with the largest proportion aged 30–34 years. The majority had a college-level education and were employed either full-time or part-time. Most participants had one child, and the target children were aged between 4 and 6 years, with a relatively even distribution across age groups. All participants reported having experience with sexuality education, while fewer had provided sexuality education to their children or reported that their children had received it (Table 3).

**Table 3:** Characteristics of participants and their children (*N* = 18).

Demographic Category		*N* (%)	
Gender	Female	16 (88.9)	
	Male	2 (11.1)	
Age (years)	≤29	2 (11.1)	(range: 28–42)
	30–34	7 (38.9)	
	35–39	6 (33.3)	
	≥40	3 (16.7)	
Educational level	High school	4 (22.2)	
	College	12 (66.7)	
	Graduate school	2 (11.1)	
Employment status	Full-time	5 (27.8)	
	Part-time	8 (44.4)	
	Unemployed	5 (27.8)	
Gender of child	Female	12 (66.7)	
	Male	6 (33.3)	
Age of target children (years)	4	5 (27.8)	
	5	6 (33.3)	
	6	7 (38.9)	
Number of children	1	12 (66.7)	
	2	5 (27.8)	
	3	1 (5.6)	
Parents’ personal experience of receiving sexuality education	Yes	18 (100)	
	No	0 (0)	
Children’s experience of sexuality education	Yes	7 (38.9)	
	No	11 (61.1)	
Experience of providing sexuality education to a child	Yes	1 (5.6)	
	No	17 (94.4)	

*Note*: ‘Personal experience of receiving sexuality education’ refers to the parents’ own past exposure to sexuality education, whereas ‘Experience of providing sexuality education’ indicates active delivery to their children.

#### 3.2.2. Interconnected Domains of Sexuality Education Among Parents of Young Children

The findings indicated that parental sexuality education is shaped by everyday situations and characterized by parents’ responses to situational demands, uncertainty, and support needs. Four interconnected domains were identified: (1) recognizing sexuality in everyday contexts, (2) adopting initial coping strategies, (3) struggling with the role–action gap, and (4) seeking structured and collaborative support (Table 4).

**Domain 1. Recognizing Sexuality in Everyday Contexts**

Parents’ experiences with sexuality education emerged not as planned instruction but as situational events triggered by daily life. Most participants reported that their awareness formed reactively rather than through intentional preparation.

**Children’s Curiosity as a Catalyst for Interaction:** Children’s natural curiosity about the body served as the primary catalyst. These moments were often experienced as fleeting instances of minor embarrassment rather than deep reflection. Inquiries about physical differences between peers or parents’ bodies were perceived as developmental milestones. One participant noted:

*“While bathing, my child asked why she didn’t have a penis; that was the first time I wondered how to explain this”*.

(P3)

Another mentioned being flustered because they *“still thought of (the child) as just a baby”* (P11). These experiences typically remained at the level of temporary recognition without leading to systematic action.

**Gendered Dynamics Shaping Early Boundaries**: Parents described differences in interactions by the child’s gender, with greater sensitivity reported in opposite-gender parent–child relationships. As children recognized physical differences, parents established boundaries, such as ensuring children remained clothed after bathing or limiting physical contact. Discontinuing shared bathing between opposite-gender parents and children was a common “first decision” regarding sexuality education.

*“As my daughter grew older, my husband and I discussed when he should stop bathing with her. That was our first conversation as a couple about sexuality education”*.

(P6)

One participant shared, “While bathing my son with his younger sister, I saw him staring intently at his sister’s anus and felt that something was necessary” (P8), leading them to “feel the need to maintain boundaries” (P8).

In domain 1, sexuality education naturally “occurs” in an unplanned state. These routine interactions initiate parental recognition and subsequent reactions but rarely lead to systematic pedagogical preparation at this stage.

**Domain 2. Adopting Initial Coping Strategies**

Parents at this stage prioritized immediate responses to resolve specific incidents rather than systematic planning. The focus remained on reactive problem-solving triggered by situational needs.

**Use of Mediated Resources for Immediate Response**: To address inquiries promptly, parents relied on mediating tools rather than direct instruction. Storybooks served as the primary resource, valued for their child-friendly accessibility and perceived expert-verified content. One participant explained:

*“I lacked the confidence to answer properly. I felt storybooks were likely written by experts, so I substituted [direct education] by reading those books”*.

(P5)

Another noted it was *“more comfortable to show a book first”* (P9), indicating that these tools functioned as a psychological buffer for parents.

**Active Information-Seeking for Situational Strategies**: Parents sought strategies by searching for similar experiences via online and offline networks. Online platforms provided a sense of relief through shared concerns, while offline conversations with acquaintances offered practical directions.

*“I searched to see if we were the only ones; I felt relieved seeing that other parents had similar concerns”*.

(P1)

Another participant mentioned listening to other mothers’ responses and *“following their lead”* (P13). This domain provided essential reference points for immediate situational management.

In domain 2, parents resolve immediate challenges through external resources. While they utilize various tools and social networks, their approach remains situation-centered and reactive, rather than proactive or planned.

**Domain 3. Struggling with the Role–Action Gap**

As children’s inquiries persisted, parents began to redefine their roles. This stage is characterized by the tension between a heightened sense of responsibility and significant psychological hurdles in execution.

**Perceived Responsibility Amidst Low Execution Priority**: Parents generally acknowledged their role as primary educators. However, amidst the broader burdens of child-rearing, sexuality education was often relegated to a low-priority task, perceived as “important but difficult to implement.” One participant noted:

*“I think it’s right for parents to provide sexuality education, but I really don’t know what to do”*.

(P4)

Another admitted it *“keeps getting pushed down the priority list”* (P10). This sense of responsibility was thus accompanied by a continuous delay in actual practice.

**Uncertainty Regarding Explanatory Scope and Methods**: Despite their perceived role, parents faced persistent uncertainty over terminology and the depth of explanation. This led to hesitation or the use of overly euphemistic, factually inaccurate narratives, often resulting in parental guilt.

*“I didn’t know how much to explain, so I… ended up explaining it like a fantasy story”*.

(P7)

Another worried whether they *“told them the wrong thing”* (P12). These narratives underscore that determining the “scope and method” remains the most daunting challenge for parents.

**Partial Reliance on External Agents as a Compensatory Strategy**: Faced with such uncertainty, parents exhibited a tendency to delegate educational roles to experts or institutions. While maintaining that parents *should* educate, they simultaneously wished for professional intervention to alleviate their burden.

*“I thought it would be better if a school or an expert did this. How great would it be if someone said they’d do it for me?”*.

(P2)

Others felt that because they were *“not good at it”* (P6), institutional teaching might be more effective. However, a few participants noted a contrasting social atmosphere, with one remarking, “When a survey was conducted on whether kindergartens should provide sexuality education, most parents said no. I was really surprised” (P8). This reflects a complementary strategy used to compensate for a lack of confidence in their own implementation capacity.

In domain 3, the recognition of responsibility does not automatically translate into action. Instead, parents remain in a cycle of hesitation, struggling with the “how-to” and increasingly seeking external validation or delegation.

**Domain 4. Seeking Structured and Collaborative Support**

Despite persistent uncertainties, parents maintained their sense of responsibility and sought structured external support to better fulfill their roles. Rather than delegating the task entirely, they aspired to become proactive educators through feasible support systems.

**Desire for Parent–Child Joint Learning Opportunities:** Parents perceived sexuality education as a joint learning process rather than a child-only activity. They viewed the family as a collective educational unit and anticipated that shared experiences would alleviate psychological burdens.

*“If I learn while experiencing it together with my child, I think I’ll naturally figure out what to do”*.

(P5)

This reflects a shift toward expanding sexuality education into a family-centered shared experience.

**Institutional Support for Strengthening Parental Capacity:** Recognizing the limits of individual responsibility, parents emphasized the need for institutional support. They believed public systems should empower parents to conduct education effectively rather than merely acting as substitutes.

*“I don’t think institutions should do everything; they should help parents do it well”*.

(P2)

This perspective positions parents as primary agents while underscoring the necessity of a systematic support framework.

**Demand for Concrete, Stage-Specific Guidelines**: Parents highlighted the absence of clear criteria regarding the “when, what, and how” of sexuality education. Comparing this to the systematic nature of infant vaccinations, they called for accessible, developmental-stage-specific guidelines.

*“I wish there were a sexuality education guideline for young children that specifies which topics should be taught at what age”*.

(P6)

Another participant (P13) noted that while infant health examinations track physical growth, they provide no guidance on sexuality, leaving parents without verified resources.

In domain 4, parents transition from situational coping to seeking a collaborative environment. They require a supportive system that enables them to act as empowered, proactive agents rather than relying on fragmented external information.

## 4. Discussion

This study was conducted to understand the response patterns and information-seeking interconnected domains of parents with young children regarding sexuality education. By integrating online post analysis and one-on-one in-depth interviews, this study examined the same phenomenon through a complementary mixed-methods approach. The digital text network analysis characterized patterns in online discourse, while the qualitative interviews provided contextual accounts of parents’ experiences. Together, the findings from the two data sources informed the integrated framework presented in Figure 3.

First, this study suggested that parental sexuality education is triggered by specific situations occurring in daily life rather than being a pre-planned educational activity. In the interviews, parents reported initiating sexuality education in response to children’s questions or body-related incidents, which aligns with previous studies suggesting that sexuality education occurs naturally through developmental curiosity and interaction [23,24,25]. The network analysis provided a complementary perspective by situating these parental experiences within a broader informational context. A community comprising “boy,” “girl,” “young child,” “mother,” “father,” “kindergarten,” and “education” was interpreted as reflecting the parent–child and educational contexts surrounding online discussions of sexuality education.

Second, parents tended to utilize immediate response strategies rather than systematic education when faced with their children’s needs. The interviews revealed that parents prioritized situational responses when lacking confidence in explanation methods. As illustrated in Figure 3, parents initially sought information through readily accessible online sources, such as internet communities, to address their immediate concerns and identify appropriate ways to respond. The network analysis provided a complementary perspective. Specifically, “sexuality education books” showed the highest degree and closeness centrality, while “recommendation” had the second-highest degree and closeness centrality. Furthermore, “sexuality education books,” “recommendation,” “curiosity,” “starting point,” “book series,” “amount of text,” “audio pen,” “concern,” and “when” formed a community that was interpreted as relating to sexuality education resources and timing. These patterns suggest that online discussions were centered on sexuality education resources, recommendations, and questions concerning when to begin sexuality education. This is consistent with previous research showing that parents tend to convey information indirectly through verified media or external resources rather than conducting direct education [23,24,25]. Previous studies have also suggested that information-seeking and sharing behaviors may differ according to the characteristics and functions of online platforms, including Q&A and community-based environments [13,26,27]. Although direct cross-platform comparisons were not made in the present study because of the unequal corpus sizes, these studies provide a broader context for understanding how the online information environment may shape parents’ access to and use of sexuality education information.

Third, although parents recognized the necessity and responsibility of sexuality education, they experienced persistent uncertainty during the implementation process. In the interviews, parents commonly reported difficulties regarding “how much to explain” and “how to deliver the information,” aligning with existing studies that show parents recognize sexuality education as a vital task but lack confidence in their own implementation capacity [8,9]. The network analysis provided a complementary perspective, as “concern,” “curiosity,” “starting point,” and “when” were included in the community related to sexuality education resources and timing. These patterns suggest that questions and concerns about when to begin sexuality education were interconnected with discussions of educational resources in the online discourse. From a practical perspective, these findings highlight the need for guidance that addresses not only the importance of sexuality education but also when and how parents can respond to children’s questions.

Fourth, parents did not limit sexuality education to an individual responsibility but recognized the need for a structural system to support it. The interviews emphasized demands for stage-specific guidelines, expert support, and parent education programs, linking back to existing discussions that sexuality education should occur within a family–institution collaborative framework [8,28,29]. The network analysis provided a broader informational context, with “kindergarten” included in the community representing parent–child and educational contexts, while “media” and “help” formed a separate community related to media and support. Although these network findings do not directly suggest parents’ demand for institutional support, they show that educational settings and support-related resources were also represented in the online discourse surrounding sexuality education. Consistent with previous research, the interview findings suggest that parents seek external support [6,8], while continuing to recognize their own roles in their children’s sexuality education. These interconnected domains—situational recognition, information-seeking, implementation uncertainty, and the need for structural support—are synthesized in Figure 3 as the interconnected domains of parental experiences in childhood sexuality education.

**Figure 3:**
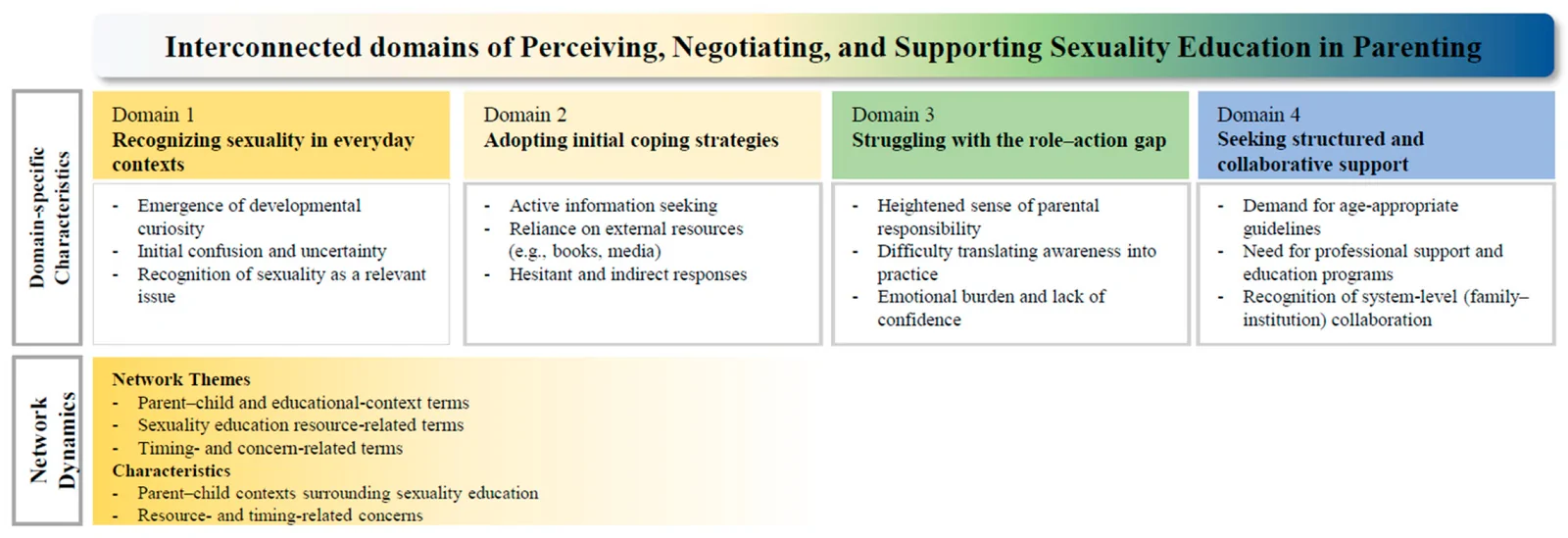
Interconnected domains of parental experiences in childhood sexuality education.

Overall, this study identified parental experiences in childhood sexuality education as comprising interconnected and potentially overlapping domains, including situational responses, mediated information strategies, implementation uncertainty, and the need for structural support, rather than a fixed linear sequence. The interview findings described how parents experienced these domains, while the network analysis identified related informational resources and structural connections in online discourse. Together, the findings highlight the importance of considering parental sexuality education within broader social, informational, and institutional contexts.

Several limitations should be considered. First, the final digital corpus was relatively small, comprising 124 online posts, and was retrieved using a single Korean search term, “성교육” (seonggyoyuk; sexuality education). This retrieval strategy may have preferentially captured posts explicitly framed as sexuality education while excluding relevant discussions expressed through alternative terms, such as body autonomy, boundaries, consent, safe or unsafe touch, digital safety, or abuse prevention. Indeed, themes related to body autonomy, consent, safe or unsafe touch, digital safety, and abuse prevention were relatively absent from the online discourse. However, this absence should be interpreted with caution, as it may reflect the limitations of the single-keyword retrieval strategy rather than a lack of parental interest in these issues. Additionally, the prominence of “sexuality education books” in the frequency and network analyses may partly reflect the lexical normalization strategy, in which contextually related book-based expressions were aggregated into a common analytical category.

Second, the numbers of posts obtained from the Q&A platform (*n* = 84) and parenting communities (*n* = 40) were unequal, which limited direct comparisons between the two sources. In addition, the demographic characteristics of online users could not be independently verified. Because the online corpus was drawn from a single national digital ecosystem, the findings may not fully reflect the experiences of parents in non-digital or offline settings, or those situated within different sociocultural and healthcare contexts.

Third, the online posts spanned from 2020 to February 2026; however, temporal changes in online discourse and information-seeking patterns were not separately examined. Moreover, online users and interview participants constituted independent samples. Therefore, the findings from the two data sources could not be linked or compared at the individual level.

Fourth, although all computational analyses were conducted using the original Korean text and translation was performed only for presentation, some linguistic or cultural nuances may have been lost in the English translation. In addition, the qualitative interview sample was predominantly female (16/18). Accordingly, paternal perspectives and opposite-gender parent–child interactions may not have been sufficiently represented, and the findings should be interpreted with caution because maternal and paternal viewpoints were not equally reflected.

Future research should employ broader search strategies and include more diverse demographic groups and digital platforms. Studies should also examine temporal changes in parental online discourse and information-seeking behaviors, include fathers and more diverse family contexts, and evaluate the effectiveness of interventions designed to support parents’ sexuality education practices.

Methodologically, this study contributes by integrating digital text network analysis with in-depth qualitative interviews, providing complementary perspectives on online information patterns and parents’ experiences. From a clinical and educational perspective, pediatric and child health professionals may serve as information navigators by providing timely, developmentally appropriate, and practical guidance to parents. At the policy level, support should extend beyond placing responsibility solely on individual parents and should include integrated institutional frameworks, parent education programs, and communication strategies tailored to different online information environments.

## 5. Conclusions

This study highlights that parental sexuality education is not a pre-planned pedagogical activity but a continuous, context-based social practice triggered by daily interactions. The findings suggest interconnected and potentially overlapping domains, from recognizing sexuality in routine life to seeking structured support, with information seeking often mediated by digital environments and educational resources. By integrating parents’ accounts with patterns identified in online discourse, this study provides an exploratory framework for understanding parental engagement and information seeking in early-childhood sexuality education. The findings may inform the development of developmentally appropriate professional guidance and accessible educational resources to support parents in navigating sexuality education in everyday situations. These implications should be considered exploratory and warrant further research to examine how clinical, educational, and institutional support may contribute to parental sexuality education practices.

## Figures and Tables

**Figure 1:**
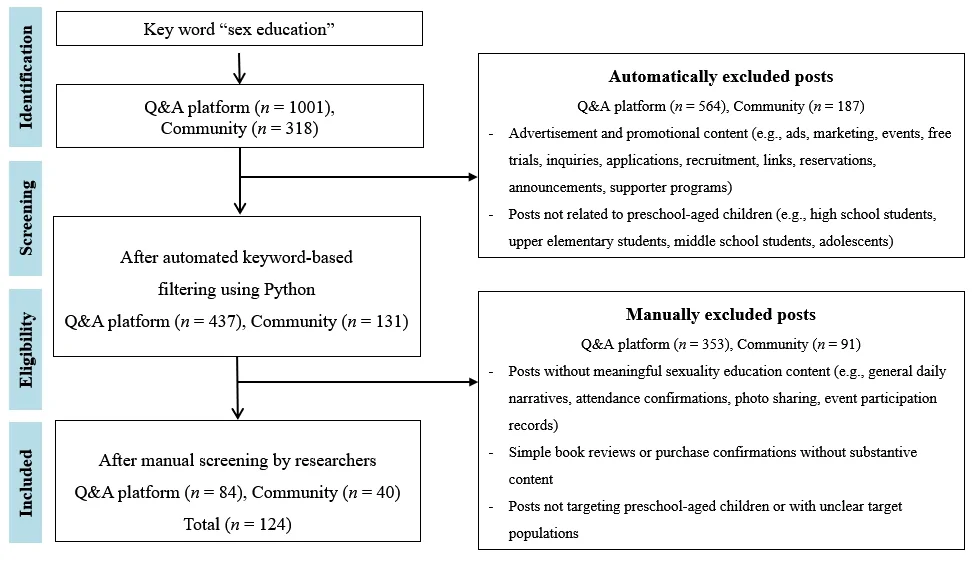
Flow diagram of online data collection and screening process.

**Figure 2:**
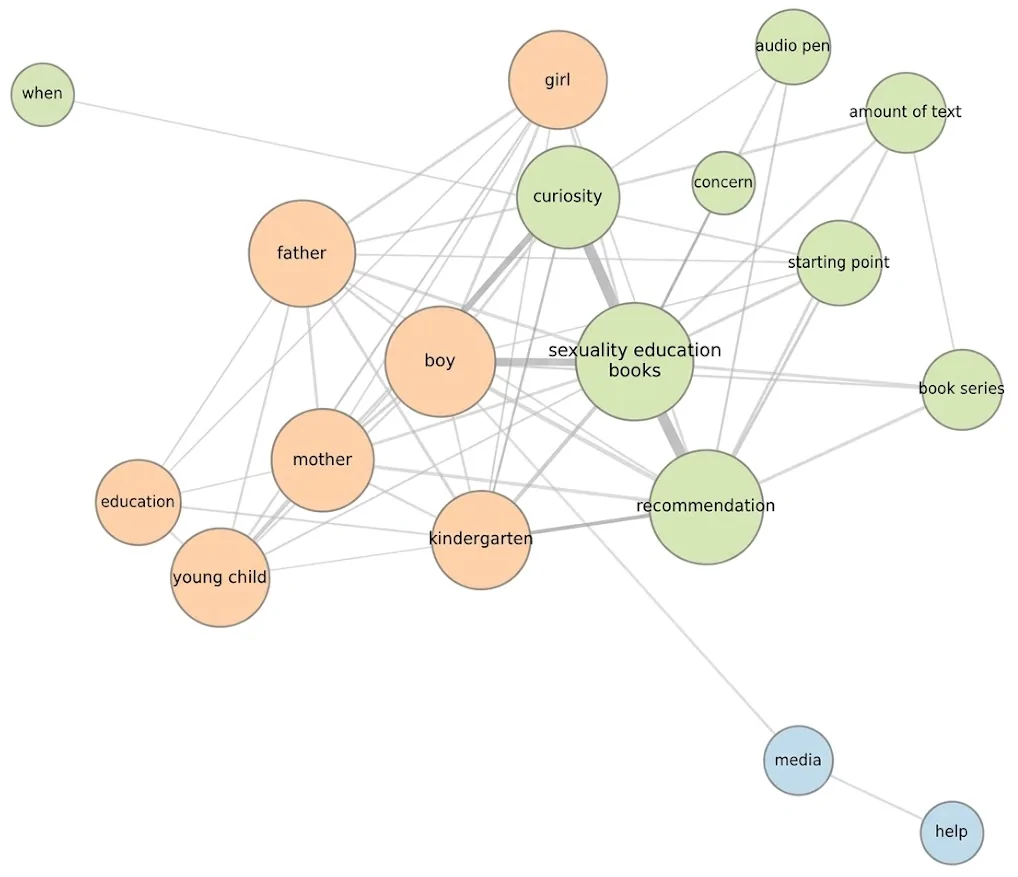
Co-occurrence network of keywords related to sexuality education. *Note*: Node colors represent the three communities identified using the Louvain community-detection algorithm. Node size represents degree centrality, and edge thickness represents sentence-level co-occurrence frequency. Node positions were manually adjusted for visual clarity and do not represent quantitative distances between keywords.

**Table 1:** Top 20 keywords related to children’s sexuality education in online posts.

Rank	Keyword (Total)	Freq.	Keyword (Community)	Freq.	Keyword (Q&A Platform)	Freq.
1	Sexuality education books	129	Sexuality education books	26	Sexuality education books	103
2	recommendation	79	recommendation	14	recommendation	65
3	boy	46	boy	10	boy	36
4	curiosity	26	media	9	kindergarten	26
5	kindergarten	26	girl	8	curiosity	21
6	girl	23	education	8	mother	20
7	mother	22	starting point	6	young child	17
8	education	22	thought	5	girl	15
9	young child	18	curiosity	5	father	14
10	father	15	when	5	education	14
11	media	13	parents	5	genitalia	9
12	starting point	12	children	4	book series	9
13	book series	11	early	3	people	8
14	people	10	concept	3	repeatedly	8
15	thought	9	exposure	3	audio pen	7
16	method	9	alone	3	age in months	7
17	help	9	news	3	home	7
18	age in months	9	stimulation	3	help	6
19	repeatedly	9	method	3	starting point	6
20	genitalia	9	help	3	behavior	6

*Note*: Freq. = Frequency.

**Table 2:** Centralities of keywords related to sexuality education from online.

Rank	Keyword	Cd	Keyword	Cb	Keyword	Cc
1	Sexuality education books	0.765	boy	0.262	Sexuality education books	0.773
2	recommendation	0.706	Sexuality education books	0.225	recommendation	0.739
3	boy	0.647	media	0.118	boy	0.739
4	father	0.588	starting point	0.118	father	0.680
5	curiosity	0.529	recommendation	0.108	curiosity	0.630
6	mother	0.529	father	0.050	mother	0.630
7	young child	0.471	curiosity	0.040	young child	0.607
8	kindergarten	0.471	mother	0.017	kindergarten	0.607
9	girl	0.471	young child	0.016	girl	0.607
10	starting point	0.294	kindergarten	0.016	starting point	0.567
11	education	0.294	girl	0.013	book series	0.515
12	book series	0.235	book series	0.006	amount of text	0.486
13	amount of text	0.235	amount of text	0.002	audio pen	0.472
14	audio pen	0.176	education	0.001	education	0.459
15	media	0.118	audio pen	0.000	media	0.459
16	concern	0.059	concern	0.000	concern	0.447
17	when	0.059	when	0.000	when	0.370
18	help	0.059	help	0.000	help	0.321

*Note*: Cd = Degree Centrality; Cb = Betweenness Centrality; Cc = Closeness Centrality.

**Table 4:** Interconnected domains of sexuality education among parents of young children.

Domain 1. Recognizing sexuality in everyday contexts	
	1-1. Children’s curiosity initiating sexuality-related interactions
	1-2. Gendered parent–child dynamics shaping boundaries and discomfort
Domain 2. Adopting initial coping strategies	
	2-1. Use of mediated resources to support immediate responses
	2-2. Active information seeking in response to situations
Domain 3. Struggling with the role–action gap	
	3-1. Acknowledging responsibility as primary educators
	3-2. Uncertainty about how to explain
	3-3. Partial reliance on external agents for guidance
Domain 4. Seeking structured and collaborative support	
	4-1. Desire for parent–child joint learning opportunities
	4-2. Institutional responsibility for strengthening parental capacity
	4-3. Demand for concrete, stage-specific educational guidelines

## Data Availability

The data that support the findings of this study are available from the corresponding author, Sujin Lee, upon reasonable request. The online textual data are derived from publicly accessible online content resources as specified in the manuscript. To protect the privacy of interview participants, qualitative transcripts are not publicly available.

## Supplementary Materials

The following supporting information can be downloaded at: https://www.mdpi.com/article/10.3390/children13101378/s1, Supplementary file S1: Interview Guides; Supplementary file S2: COREQ; Supplementary file S3: Privacy-preserving materials; Supplementary file S4: Python Code for Preprocessing and Online Text Network Analysis.

## Abbreviations

The following abbreviations are used in this manuscript:

BZgA	Regional Office for Europe and the Federal Centre for Health Education
SNA	Social Network Analysis
UNESCO	United Nations Educational, Scientific and Cultural Organization
WHO	World Health Organization
