# Supplementary material for: From Awareness to Practice: How Parents Seek Support for Children’s Sexuality Education
Source: Children (Basel). 2026 Oct 11;13(10):1378. doi: 10.3390/children13101378 (PMC13703016; doi:10.3390/children13101378)
Supplement: Supplementary file 1 [file children-13-01378-s001.zip › Supplementary S1_Interview Guide.pdf]

# Semi-Structured Interview Guide

## I. Introduction & Warm-up

- Could you briefly introduce your child/children and share your general experiences as a parent?
- How do you usually perceive or think about children's growth, bodily awareness, and sex education in your daily life?

## II. Core Semi-Structured Questions

1. **Perceptions and Experiences of Children's Sexual Health & Education:**
    - What are your thoughts or general attitudes toward introducing sex education to young children? Have you ever tried discussing related topics with your child?
    - Could you share specific situations, questions, or moments from your child (e.g., questions about where babies come from, body curiosity, or boundaries) that made you feel challenged or concerned?
2. **Needs, Barriers, and Information Seeking:**
    - Where do you usually look for information or guidance regarding child sex education or reproductive health? What difficulties or barriers do you encounter when trying to access reliable information?
    - What specific information, educational content, or professional support do you feel is most lacking or urgently needed for parents?
3. **Preferences for Interventions or Platforms:**
    - If an educational program, platform, or coaching tool (e.g., mobile-based or community-based) were developed to support parents in providing sex education, what features, content, or formats would you find most helpful?

## III. Closing

- Is there anything else regarding your experiences, concerns, or expectations about children's sex education that we haven't discussed yet?
- Thank you for your valuable time and contribution to this study.
