# Supplementary material for: From Awareness to Practice: How Parents Seek Support for Children’s Sexuality Education
Source: Children (Basel). 2026 Oct 11;13(10):1378. doi: 10.3390/children13101378 (PMC13703016; doi:10.3390/children13101378)
Supplement: Supplementary file 1 [file children-13-01378-s001.zip › Supplementary S2_COREQ.pdf]

Consolidated criteria for reporting qualitative studies (COREQ): 32-item checklist

From Awareness to Practice: How Parents Seek Support for Children's Sexuality Education

No. Item	Guide questions/description	Reported on Page #
**Domain 1: Research team and reflexivity**		
Personal Characteristics		
1. Inter viewer/facilitator	Which author/s conducted the interview or focus group?	4
2. Credentials	What were the researcher's credentials? E.g. PhD, MD	4
3. Occupation	What was their occupation at the time of the study?	4
4. Gender	Was the researcher male or female?	4
5. Experience and training	What experience or training did the researcher have?	4
Relationship with participants		
6. Relationship established	Was a relationship established prior to study commencement?	4
7. Participant knowledge of the interviewer	What did the participants know about the researcher? e.g. personal goals, reasons for doing the research	4-5
8. Interviewer characteristics	What characteristics were reported about the inter viewer/facilitator? e.g. Bias, assumptions, reasons and interests in the research topic	5

**Domain 2: study design**		
Theoretical framework		
9. Methodological orientation and Theory	What methodological orientation was stated to underpin the study? e.g. grounded theory, discourse analysis, ethnography, phenomenology, content analysis	3
Participant selection		
10. Sampling	How were participants selected? e.g. purposive, convenience, consecutive, snowball	3
11. Method of approach	How were participants approached? e.g. face-to-face, telephone, mail, email	3
12. Sample size	How many participants were in the study?	5
13. Non-participation	How many people refused to participate or dropped out? Reasons?	5
Setting		
14. Setting of data collection	Where was the data collected? e.g. home, clinic, workplace	5
15. Presence of non-participants	Was anyone else present besides the participants and researchers?	5
16. Description of sample	What are the important characteristics of the sample? e.g. demographic data, date	9
Data collection		
17. Interview guide	Were questions, prompts, guides provided by the authors? Was it pilot tested?	Supplementary Materials: Interview Guides
18. Repeat interviews	Were repeat inter views carried out? If yes, how many?	5
19. Audio/visual recording	Did the research use audio or visual recording to collect the data?	5
20. Field notes	Were field notes made during and/or after the interview or focus group?	5
21. Duration	What was the duration of the interviews or focus group?	5
22. Data saturation	Was data saturation discussed?	6
23. Transcripts returned	Were transcripts returned to participants for comment and/or correction?	Not reported
**Domain 3: analysis and findings**		
Data analysis		
24. Number of data coders	How many data coders coded the data?	6
25. Description of the coding tree	Did authors provide a description of the coding tree?	10-11
26. Derivation of themes	Were themes identified in advance or derived from	3

	the data?	
27. Software	What software, if applicable, was used to manage the data?	6
28. Participant checking	Did participants provide feedback on the findings?	Not reported
Reporting		
29. Quotations presented	Were participant quotations presented to illustrate the themes/findings? Was each quotation identified? e.g. participant number	10-13
30. Data and findings consistent	Was there consistency between the data presented and the findings?	10-13
31. Clarity of major themes	Were major themes clearly presented in the findings?	10-11
32. Clarity of minor themes	Is there a description of diverse cases or discussion of minor themes?	6
