# Supplementary material for: From Awareness to Practice: How Parents Seek Support for Children’s Sexuality Education
Source: Children (Basel). 2026 Oct 11;13(10):1378. doi: 10.3390/children13101378 (PMC13703016; doi:10.3390/children13101378)
Supplement: Supplementary file 1 [file children-13-01378-s001.zip › Supplementary S4_Python Code for Preprocessing and Online Text Network Analysis.pdf]

Supplementary File S4

Python Code for Preprocessing and Online Text Network Analysis

This supplementary file provides the privacy-preserving Python code used for the preprocessing and computational analysis of the online corpus. The code covers Korean text preprocessing, document-level TF-IDF calculation, sentence-level co-occurrence analysis, network construction, centrality analysis, and Louvain community detection. This code reproduces the analytical procedures performed after selection of the final analytical corpus and begins with the finalized datasets comprising 84 Q&A posts, 40 community posts, and 124 combined posts. It does not reproduce the initial web-crawling or automated keyword-based screening procedures used during corpus construction.

No original online posts, verbatim text, URLs, usernames or user IDs, post-level metadata, document-level tokenized text, or document-level TF-IDF data are included in this file.

Software environment: Python 3.13.15; KoNLPy 0.6.0 (Okt); pandas 2.2.3; NetworkX 3.6.1; scikit-learn 1.6.1.

Analysis Code

```
"""
Supplementary File S4
Privacy-preserving Python script for Korean online text and network analysis.

This script contains the preprocessing and analytical procedures used in the
final analysis. No original posts, verbatim text, URLs, usernames/user IDs,
post-level metadata, or document-level analytical outputs are embedded.

Expected inputs
---------------
Three .xlsx files supplied locally by an authorized researcher:
1) Q&A corpus: 84 rows
2) Community corpus: 40 rows
3) Combined corpus: 124 rows

Each file must contain a title column and a body/content column.
The script writes aggregate analytical outputs only.

Software used in the final analysis
-----------------------------------
Python 3.13.15
KoNLPy 0.6.0 (Okt)
pandas 2.2.3
NetworkX 3.6.1
scikit-learn 1.6.1
"""

import re
import sys
from collections import Counter
from itertools import combinations
from pathlib import Path

import numpy as np
import pandas as pd
import networkx as nx
import sklearn
import konlpy
from konlpy.tag import Okt
from sklearn.feature_extraction.text import TfidfVectorizer


# ---------------------------------------------------------------------------
# 1. PARAMETERS
# ---------------------------------------------------------------------------

EDGE_THRESHOLD = 2
TOP_N_TFIDF = 20
LOUVAIN_RESOLUTION = 1.0
LOUVAIN_SEED = 42

BOOK_PATTERNS = sorted([
```

```
    "성 교육 동화책", "성교육 동화책", "성교육동화책",
    "성 교육 동화", "성교육 동화", "성교육동화",
    "성 교육 그림책", "성교육 그림책", "성교육그림책",
    "성 교육 전집", "성교육 전집", "성교육전집",
    "성 교육 도서", "성교육 도서", "성교육도서",
    "성 교육 책", "성교육 책", "성교육책"
], key=len, reverse=True)

SAYPEN_PATTERNS = ["세이 펜", "세이펜"]

REPLACEMENTS = dict(sorted({
    "남자 아이": "남아", "남자아이": "남아", "남자 애": "남아",
    "여자 아이": "여아", "여자아이": "여아", "여자 애": "여아",
    "동화 책": "동화책", "그림 책": "그림책", "그림 동화": "그림동화",
    "유 튜브": "유튜브", "스마트 폰": "스마트폰",
    "성 교육": "성교육", "성 병": "성병", "성 차별": "성차별"
}.items(), key=lambda x: len(x[0]), reverse=True))

SYNONYMS = {
    "동화": "성교육도서", "동화책": "성교육도서",
    "그림책": "성교육도서", "그림동화": "성교육도서",
    "부모님": "부모",
    "유튜브": "미디어", "유투브": "미디어", "티비": "미디어",
    "영상": "미디어", "스마트폰": "미디어", "핸드폰": "미디어",
    "남자": "남아", "아들": "남아",
    "여자": "여아", "딸": "여아", "딸아이": "여아",
    "호기심": "궁금", "관심": "궁금", "궁금증": "궁금"
}

STOPWORDS = {
    "오늘","정말","너무","진짜","조금","많이","그냥",
    "우리","저희","요즘","이제","성교육",
    "아이","유아","어린이",
    "관련","정도","부분","경우","내용","대해","대한","위해",
    "혹시","부탁","알려","해주세요","보고","보기","참고",
    "답변","하나요","는걸","때문",
    "다른","이번","올해","살이","슬슬"
}


# ---------------------------------------------------------------------------
# 2. INPUT HELPERS
# ---------------------------------------------------------------------------

def detect_text_columns(df):
    title_candidates = ["제목", "title", "Title"]
    body_candidates = [
        "본문", "description", "Description", "내용",
        "body", "Body", "text", "Text"
    ]
    title_col = next((c for c in title_candidates if c in df.columns), None)
    body_col = next((c for c in body_candidates if c in df.columns), None)
    if title_col is None or body_col is None:
        raise KeyError("Could not identify title/body columns.")
    return title_col, body_col


def standardize_dataframe(df, platform_name):
    title_col, body_col = detect_text_columns(df)
    out = pd.DataFrame()
    out["title"] = df[title_col].fillna("").astype(str)
    out["body"] = df[body_col].fillna("").astype(str)
    out["text"] = out["title"] + " " + out["body"]
    out["platform"] = platform_name
    return out
```

```
# ---------------------------------------------------------------------------
# 3. PREPROCESSING
# ---------------------------------------------------------------------------

okt = Okt()


def extract_book_resources(text):
    working = str(text)
    count = 0
    for pattern in BOOK_PATTERNS:
        n = working.count(pattern)
        if n:
            count += n
            working = working.replace(pattern, " ")
    return working, count


def extract_saypen(text):
    working = str(text)
    count = 0
    for pattern in SAYPEN_PATTERNS:
        n = working.count(pattern)
        if n:
            count += n
            working = working.replace(pattern, " ")
    return working, count


def tokenize_text(text):
    text, book_count = extract_book_resources(str(text))
    text, saypen_count = extract_saypen(text)

    for old, new in REPLACEMENTS.items():
        text = text.replace(old, new)

    text = re.sub(r"[^가-힣 ]", " ", text)
    text = re.sub(r"\s+", " ", text).strip()

    nouns = okt.nouns(text)
    tokens = []
    for word in nouns:
        normalized = SYNONYMS.get(word, word)
        if len(normalized) <= 1:
            continue
        if normalized in STOPWORDS:
            continue
        tokens.append(normalized)

    tokens.extend(["성교육도서"] * book_count)
    tokens.extend(["세이펜"] * saypen_count)
    return tokens


def corpus_counter(token_series):
    counter = Counter()
    for tokens in token_series:
        counter.update(tokens)
    return counter


# ---------------------------------------------------------------------------
# 4. AGGREGATE TERM FREQUENCY
# ---------------------------------------------------------------------------

def make_frequency_table(counter):
    df = pd.DataFrame(counter.most_common(), columns=["word", "frequency"])
    df.insert(0, "rank", range(1, len(df) + 1))
    return df


# ---------------------------------------------------------------------------
# 5. DOCUMENT-LEVEL TF-IDF
# ---------------------------------------------------------------------------

def calculate_mean_tfidf(combined):
    tokenized_documents = combined["tokens"].apply(lambda x: " ".join(x))
    vectorizer = TfidfVectorizer(
```

```python
        tokenizer=str.split,
        preprocessor=None,
        token_pattern=None,
        lowercase=False
    )
    matrix = vectorizer.fit_transform(tokenized_documents)
    terms = vectorizer.get_feature_names_out()
    means = np.asarray(matrix.mean(axis=0)).ravel()

    result = pd.DataFrame({"word": terms, "mean_tfidf": means})
    result = result.sort_values(
        ["mean_tfidf", "word"], ascending=[False, True]
    ).reset_index(drop=True)
    result.insert(0, "rank", range(1, len(result) + 1))
    return result


# ---------------------------------------------------------------------------
# 6. SENTENCE-LEVEL CO-OCCURRENCE
# ---------------------------------------------------------------------------

def split_sentences(text):
    return [
        s.strip()
        for s in re.split(r"[.!?\n\r]+", str(text))
        if s.strip()
    ]


def calculate_cooccurrence(combined, candidate_terms):
    candidate_terms = set(candidate_terms)
    counter = Counter()

    for text in combined["text"]:
        for sentence in split_sentences(text):
            tokens = tokenize_text(sentence)
            selected = [t for t in tokens if t in candidate_terms]
            # Repeated occurrences of the same term in one sentence count once.
            unique_terms = list(dict.fromkeys(selected))
            if len(unique_terms) >= 2:
                for w1, w2 in combinations(sorted(unique_terms), 2):
                    counter[(w1, w2)] += 1

    rows = [
        {"word1": pair[0], "word2": pair[1], "weight": weight}
        for pair, weight in counter.items()
    ]
    df = pd.DataFrame(rows)
    if len(df):
        df = df.sort_values(
            ["weight", "word1", "word2"],
            ascending=[False, True, True]
        ).reset_index(drop=True)
    return df


# ---------------------------------------------------------------------------
# 7. NETWORK, CENTRALITY, AND LOUVAIN COMMUNITIES
# ---------------------------------------------------------------------------

def build_network(cooccurrence_df):
    retained = cooccurrence_df[
        cooccurrence_df["weight"] >= EDGE_THRESHOLD
    ].copy().reset_index(drop=True)

    graph = nx.Graph()
    for _, row in retained.iterrows():
        graph.add_edge(
            row["word1"], row["word2"], weight=float(row["weight"])
        )
    return graph, retained


def calculate_centrality(graph):
    # Centrality is calculated on the unweighted retained topology.
    degree = nx.degree_centrality(graph)
    betweenness = nx.betweenness_centrality(
        graph, weight=None, normalized=True
    )
    closeness = nx.closeness_centrality(graph)
```

```
    rows = []
    for node in graph.nodes():
        rows.append({
            "word": node,
            "degree_centrality": degree[node],
            "betweenness_centrality": betweenness[node],
            "closeness_centrality": closeness[node]
        })

    df = pd.DataFrame(rows).sort_values(
        ["degree_centrality", "betweenness_centrality",
         "closeness_centrality", "word"],
        ascending=[False, False, False, True]
    ).reset_index(drop=True)
    df.insert(0, "rank", range(1, len(df) + 1))
    return df


def detect_louvain_communities(graph):
    communities = nx.community.louvain_communities(
        graph,
        weight="weight",
        resolution=LOUVAIN_RESOLUTION,
        seed=LOUVAIN_SEED
    )
    communities = sorted(
        communities,
        key=lambda community: (-len(community), sorted(community))
    )

    rows = []
    for number, community in enumerate(communities, start=1):
        for term in sorted(community):
            rows.append({"community": number, "word": term})

    modularity = nx.community.modularity(
        graph, communities, weight="weight"
    )
    return pd.DataFrame(rows), modularity


def cooccurrence_matrix(graph, node_order):
    matrix = pd.DataFrame(0, index=node_order, columns=node_order, dtype=int)
    for u, v, data in graph.edges(data=True):
        weight = int(data["weight"])
        matrix.loc[u, v] = weight
        matrix.loc[v, u] = weight
    matrix.index.name = "word"
    return matrix


# ---------------------------------------------------------------------------
# 8. RUN ANALYSIS AND EXPORT AGGREGATE RESULTS ONLY
# ---------------------------------------------------------------------------

def run_analysis(qa_path, community_path, combined_path, output_path):
    qa_raw = pd.read_excel(qa_path)
    community_raw = pd.read_excel(community_path)
    combined_raw = pd.read_excel(combined_path)

    if len(qa_raw) != 84 or len(community_raw) != 40 or len(combined_raw) != 124:
        raise ValueError("Unexpected corpus size.")

    qa = standardize_dataframe(qa_raw, "Q&A")
    community = standardize_dataframe(community_raw, "Community")
    combined = standardize_dataframe(combined_raw, "Combined")

    qa["tokens"] = qa["text"].apply(tokenize_text)
    community["tokens"] = community["text"].apply(tokenize_text)
    combined["tokens"] = combined["text"].apply(tokenize_text)

    freq_qa = make_frequency_table(corpus_counter(qa["tokens"]))
    freq_community = make_frequency_table(corpus_counter(community["tokens"]))
    freq_combined = make_frequency_table(corpus_counter(combined["tokens"]))

    tfidf_all = calculate_mean_tfidf(combined)
    tfidf_top20 = tfidf_all.head(TOP_N_TFIDF).copy()
    candidate_terms = tfidf_top20["word"].tolist()

    cooccurrence_all = calculate_cooccurrence(combined, candidate_terms)
    graph, edge_list = build_network(cooccurrence_all)
```

```python
    centrality = calculate_centrality(graph)
    community_membership, modularity = detect_louvain_communities(graph)
    matrix = cooccurrence_matrix(graph, centrality["word"].tolist())

    summary = pd.DataFrame([
        ["Number of documents", len(combined)],
        ["Q&A documents", len(qa)],
        ["Community documents", len(community)],
        ["TF-IDF candidate terms", TOP_N_TFIDF],
        ["Co-occurrence unit", "Sentence"],
        ["Network type", "Weighted, undirected"],
        ["Edge threshold", ">= 2 sentence-level co-occurrences"],
        ["Network nodes", graph.number_of_nodes()],
        ["Network edges", graph.number_of_edges()],
        ["Connected components", nx.number_connected_components(graph)],
        ["Network density", nx.density(graph)],
        ["Louvain resolution", LOUVAIN_RESOLUTION],
        ["Weighted modularity", modularity],
    ], columns=["measure", "value"])

    # IMPORTANT: only aggregate outputs are exported.
    # No original text, sentence audit, URLs, identifiers, or document-level
    # token/TF-IDF matrices are written to the output workbook.
    with pd.ExcelWriter(output_path, engine="openpyxl") as writer:
        freq_combined.to_excel(writer, "Frequency_Combined", index=False)
        freq_qa.to_excel(writer, "Frequency_QA", index=False)
        freq_community.to_excel(writer, "Frequency_Community", index=False)
        tfidf_top20.to_excel(writer, "Mean_TFIDF_Top20", index=False)
        matrix.to_excel(writer, "Cooccurrence_Matrix")
        edge_list.to_excel(writer, "Network_Edge_List", index=False)
        centrality.to_excel(writer, "Centrality", index=False)
        community_membership.to_excel(
            writer, "Louvain_Communities", index=False
        )
        summary.to_excel(writer, "Network_Summary", index=False)


if __name__ == "__main__":
    if len(sys.argv) != 5:
        raise SystemExit(
            "Usage: python Supplementary_File_S4_Analysis_Script.py "
            "<qa.xlsx> <community.xlsx> <combined.xlsx> <output.xlsx>"
        )

    run_analysis(
        Path(sys.argv[1]),
        Path(sys.argv[2]),
        Path(sys.argv[3]),
        Path(sys.argv[4])
    )
```
